# A cluster randomised controlled trial of educational prompts in diabetes care: study protocol

**DOI:** 10.1186/1748-5908-2-22

**Published:** 2007-07-24

**Authors:** Robbie Foy, Gillian Hawthorne, Ian Gibb, Martin P Eccles, Nick Steen, Susan Hrisos, Trevor White, Bernard L Croal, Jeremy M Grimshaw

**Affiliations:** 1 Institute for Health and Society, Newcastle University, 21 Claremont Place, Newcastle upon Tyne, NE2 4AA, UK; 2 Newcastle Primary Care Trust, Benfield Road, Newcastle Upon Tyne, NE6 4PF, UK; 3 Newcastle Hospitals NHS Trust, Queen Victoria Road, Newcastle Upon Tyne, NE1 4LP, UK; 4 Grampian University Hospitals Trust, Foresterhill, Aberdeen, AB25 2ZN, UK; 5 Ottawa Health Research Institute, 725 Parkdale Avenue, Ottawa, ON K1Y 4E9, Canada

## Abstract

**Background:**

Laboratory services have a central role in supporting screening, diagnosis, and management of patients. The increase in chronic disease management in primary care for conditions such as diabetes mellitus requires regular monitoring of patients' biochemical parameters. This process offers a route for improving the quality of care that patients receive by using test results as a vehicle for delivering educational messages as well as the test result itself.

**Aim:**

To develop and evaluate the effectiveness of a quality improvement initiative to improve the care of patients with diabetes using test report reminders.

**Design:**

A programme of four cluster randomised controlled trials within one population of general practices.

**Participants:**

General practices in Newcastle-upon-Tyne, UK.

**Intervention:**

Brief educational messages added to paper and electronic general practice laboratory test reports introduced over two phases. Phase One messages, attached to Haemoglobin A1c (HbA1c) reports, targeted glycaemic and cholesterol control. Phase Two messages, attached to albumin:creatinine ratio (ACR) reports, targeted blood pressure (BP) control and foot inspection.

**Outcomes:**

General practice mean levels of HbA1c and cholesterol (Phase One) and diastolic and systolic BP and proportions of patients having undergone foot inspections (Phase Two); number of tests requested.

**Trial registration:**

Current Controlled Trial ISRCTN2186314.

## Background

There is broad, international agreement over what constitutes high quality health care for people with diabetes [1]. In the UK (UK), this is enshrined in a National Service Framework for people with diabetes, and a series of clinical practice guidelines from the National Institute for Health and Clinical Excellence (NICE). However, despite this increasing clarity on desirable care, there is evidence of suboptimal performance [2].

It is well known that guidelines do not implement themselves, and that active strategies are needed to enhance their uptake. While there are a range of means for approaching this, one that has the potential advantages of feasibility and simplicity is the attachment of educational messages to the results of laboratory tests ordered in general practice.

Laboratory services have a central role in supporting screening, diagnosis, and management of patients and represent a significant expenditure for the UK National Health Service (NHS). Locally, the number of general practitioner (GP) laboratory requests is increasing by 5% each year, and such requests currently account for approximately 25% of all tests performed by the Newcastle Hospitals NHS Trust. In relation to diabetes, over a six-month period from January 2004, GP requests accounted for 35% of all Haemoglobin A1c (HbA1c) requests processed and for 52% of all cholesterol (1,557 and 5,769 per month, respectively). There are several factors that may have contributed to this increase in demand (for example, shifts in the delivery of healthcare to primary care, the impact of clinical guidelines and protocols). Across the UK, the recent major increase in requests for several tests (including HbA1c and cholesterol) has been related to the introduction of a new GP contract that ties financial incentives to performance, particularly around chronic disease management [3,4].

Although it is difficult to judge whether this trend represents an increase in clinically appropriate practice, it has highlighted the need for evidence on how to influence test-ordering behaviour and potential impact – in terms of volume – of using test results to influence other clinical behaviours.

Available interventions to change test-ordering-related behaviours have not been systematically evaluated in a NHS setting. There is one relevant systematic review of interventions to improve test-ordering behaviour. A review of 49 studies concluded that audit and feedback of test-requesting patterns, educational messages, changes of request form, and guidelines were all effective in changing test-ordering behaviour [5]. However, the studies reviewed were of variable methodological quality, with only eight of 49 using randomisation. A randomised trial by members of this group (ME, JG and NS) demonstrated that educational reminders printed on X-ray reports returned to GPs reduced knee and lumbar spine X-ray requests by 20% (relative reduction) [6], and that this effect was sustained over 12 months of the intervention [7]. Another trial (by BC, JG and JG) indicated that enhanced feedback of requesting rates and brief educational reminder messages, alone and in combination, are effective strategies for reducing test requesting in primary care [8].

All but one of these studies were concerned with either decreasing the overall volume of tests ordered or decreasing the number of inappropriate tests ordered. Less is known about the effectiveness of the methods in increasing appropriate behaviour; the one study examining this was a trial of adding guidelines to patients' records conducted in a hospital setting. Subsequent to the above review, an NHS-based study of investigation and treatment of iron deficiency anaemia found that a simple message on a haematology test result increased the appropriate prescription of oral iron, but did not increase the appropriate investigation of patients [9]. The broader evidence base on the use of educational reminders suggests that they are more consistently effective compared with other professional behaviour change strategies [10]. However, their effects may be related to the types and complexities of targeted behaviours.

A systematic review addressing the effects of quality improvement strategies for type II diabetes on glycaemic control suggested that changes in the structure and process of care are effective. However, problems with classifying complex interventions and the relative lack of high quality studies indicate the need for further work to identify effective and sustainable ways of improving diabetes care [11]. This evaluation will focus on the effects of educational reminders on both test-ordering behaviour. Its aims are to increase appropriate test-ordering and related clinical practice, and to improve the outcomes of diabetes management. A completed checklist detailing adherence of this report to CONSORT criteria for randomised controlled trials is available as Additional File 1.

## Aims

1. To establish a quality improvement initiative to improve the quality of care for patients with diabetes involving the phased introduction of test report messages.

2. To evaluate the effectiveness of each of four test report messages within one population of general practices

## Methods

### Participants

All 38 general practices in Newcastle that mainly used the laboratory services of the Newcastle Hospitals Trust (Royal Victoria Infirmary, Newcastle General Hospital, and Freeman Hospital) at the point of the study start date were eligible for participation. The planned interventions were viewed as part of intended normal service development.

General practices received a postal invitation to participate in the study. This invitation, addressed to the practice manager, included a study information sheet in the form of a cover letter and two copies of a consent form. The manager was asked to share these materials with members of the practice team and provide signed consent if the practice wished to participate (signed either by the senior partner or the practice manager). This was an appropriate level for seeking consent given that the practices would also represent the unit of randomisation. Practices wishing further details and clarification were offered a practice visit to explain more about the study.

Following this 'opt-in' approach to gain consent from general practices, 35 practices consented to participate; only two declined and one practice was about to close.

### Interventions

The precise details of the intervention (wording and clinical content) were developed by a multi-disciplinary group, including representatives of primary care, secondary care, laboratory services, and the research team. The content of the messages is congruent with the local diabetes guideline and is evidence-based. The messages give succinct educational information regarding appropriate patient management (Table 1). The interventions were introduced in two phases, with Phase One demonstrating intervention feasibility and justifying continued funding for Phase Two.

**Table 1:** Conditions and content of brief educational messages attached to test reports.

**Study phase**	**Type and attachment of message**	**Content of message**
Phase One	Unconditional; attached to all cholesterol reports	For type 2 diabetes &*age *≥ *40 yrs*: on simvastatin 40 mg? See [local guideline] for detail and exclusions
	Conditional; attached to all HbA1c reports	If HbA1c < 6.5%
		"Within target for type 2 diabetes"
		If HbA1c 6.5 – 7.0%
		"For type 2 diabetes, consider increasing oral therapy"
		If HbA1c 7.0–8.0%
		"If type 2 diabetes: on max oral therapy, *e.g*., Metformin 1G BD + gliclazide 160 mg BD?"
		If HbA1c > 8.0%
		"If type 2 diabetes, consider insulin if on max oral Rx, *e.g*., Metformin 1G BD + gliclazide 160 mg BD"
Phase Two	Unconditional; attached to all albumin: creatinine ratio (ACR) test reports	Newcastle Diabetes Guideline Footcare: all patients annual review of sensation, pulses, footwear education
	Conditional; attached to all ACR test reports	If ACR above 2.5
		If confirmed microalbuminuria: aim for BP < 130/80 in type 2 diabetes
		If ACR below 2.5
		If no microalbuminuria: aim for BP control < 140/80 in type 2 diabetes

Phase One messages are attached to electronic and paper test reports of HbA1c. From general practice, almost all requests for HbA1c will relate to patients with diabetes. The messages are of two types. The first message relates to glycaemic control, is conditional on the HbA1c level, and gives general advice about appropriate treatment. The second message (again on an HbA1c test result form) gives a non-specific message relating to the treatment of hypercholesterolaemia in patients with diabetes. This started in December 2005.

Phase Two messages are attached to ACR test reports and are also of two types. The first message relates to foot inspection and is attached to all ACR test reports. The second message relates to blood pressure control, is conditional on the ACR level, and gives advice on target blood pressure (BP) levels for patients with and without a diagnosis of microalbuminuria. This started in October 2006.

The clinical foci of the interventions (*e.g*., glycaemic control) described here were selected because of their clinical importance. We also considered whether there would be scope for measurable improvements in patient outcomes and relevant outcome data available.

### Design

This programme comprises four cluster randomised controlled trials (one for each of the four interventions) with general practices as the unit of randomisation. It is important that the study uses a randomised design because there will inevitably be other initiatives relating to diabetes that will occur during the time of the study. It is only by using a randomised design that any observed effects can be confidently attributed to the interventions.

The availability of practice-level data used to measure performance for the Quality Outcomes Framework (QOF) allowed us to stratify randomisation. For Phase One, stratification was according to both the number of diabetic patients per practice and the QOF score (proportion of diabetic patients achieving HbA1c of 7.4 or less). For Phase Two, stratification was according to both the proportion of patients with a record of foot examination and the proportion of patients with a record or blood pressure of 145/85 or less. Therefore, practices, stratified by performance, were randomly allocated to each intervention independently. Randomisations were conducted independently by a statistician using numbers randomly generated by computer.

### Phase One

In the first randomisation, practices were allocated to receive the glycaemic educational messages or control (no glycaemic educational messages). In the second randomisation, practices were allocated to receive the cholesterol educational messages or control (no cholesterol educational messages).

### Phase Two

After nine months, practices were re-randomised and again assigned to each of two interventions. In the first randomisation, practices were allocated to receive the foot inspection reminder message or control (no foot inspection reminder message). In the second randomisation, practices were allocated to receive the blood pressure educational messages or control (no blood pressure messages).

The four randomisations allow comparisons of the separate effects of the four educational message interventions. The study is not powered to compare the effects of various combinations of intervention.

Following recruitment and randomisation, two further participating practices merged, bringing the number of enrolled practices to 34. These practices are now receiving the educational messages according to their respective randomisations.

### Outcomes

For Phase One, the two primary outcomes will be the general practice mean levels of HbA1c and cholesterol. We hypothesise that in practices receiving the glycaemic and cholesterol messages, compared to those practices not, the mean HbA1c and cholesterol values will be lower, respectively. Secondary outcomes for Phase One include the number of HbA1c and cholesterol tests requested (standardised for practice size) and the proportions of patients within each practice meeting QOF targets for glycaemic and cholesterol control.

For Phase Two, the primary outcomes will be the general practice mean levels of diastolic and systolic BP, and proportions of patients having undergone foot inspections. We hypothesize that in practices receiving the blood pressure messages compared to those practices not, the mean diastolic and systolic blood pressure values will be lower; and in those receiving foot inspections messages, the proportions of patients receiving a foot inspection will be higher. Secondary outcomes for Phase Two include: the proportions of patients in each general practice within target levels for blood pressure control according to the presence or otherwise of recorded microalbuminuria (which requires tighter BP control); the overall number of ACR tests requested (standardised for practice size); and the number of ACR tests requested (standardised for practice size) for patients with and without a diagnosis of microalbuminuria.

### Data collection

There are three main potential sources of data to assess the effectiveness of the messages: general practice-held data; centrally-held and publicly available QOF data; and centrally-held laboratory data. We shall only use the QOF and laboratory data if we are unable to collect practice-held data from all or most practices. However, the plans for all forms of data collection are discussed below.

### Practice-held data

General practices routinely collect standardised patient data that contribute to the calculation of QOF scores. Although 'exception reporting' allows practices to exclude patients with diabetes who consistently do not attend for care, practice data are scrutinised for completeness by local Primary Care Trusts (PCTs). These QOF data will be used to measure the primary outcomes for the trial,*i.e*., practice mean levels of HbA1c, cholesterol, diastolic and systolic BP, and proportions of patients having undergone foot inspections. Target levels for BP control depend on whether microalbuminuria is present. These data will also allow us to identify the subgroup of patients with a diagnosis of microalbuminuria so that their levels of BP control can be determined.

Collection and processing of these data will be undertaken on our behalf by Newcastle PCT, and all data will be anonymised before being transferred to the research team. Additional consent will be sought from practices for this data collection. To minimise the impact on practices, pre- and post-intervention outcome data will be collected during a single visit to individual practices. Data collection periods will cover 24 month pre-intervention periods (to provide baseline values), and up to 18 months post-intervention for phase one and eight months for phase two.

### Quality outcomes framework

The general practice-level data now routinely collected and publicly reported for QOF monitoring offers a potential and easily accessible means of monitoring and evaluating the impact of quality improvement strategies. Although these measures may be less sensitive to change than the main study outcomes, any impact will increase the utility of the intervention to general practices (*i.e*., if it is seen to improve scores and hence incomes for practices). We will collect relevant QOF data for the time periods corresponding to the study intervention periods.

### Laboratory-held data

Because the laboratory currently cannot routinely identify patients with diabetes, we will use HbA1c test requests as a marker for patients with diabetes. From general practice almost all requests for HbA1c relate to patients with diabetes. We will identify cholesterol test requests carried out in patients who have had HbA1c tests. This data linkage activity will be done as a manual interrogation of the laboratory computer information system by an NHS laboratory scientist. All data are anonymised at source before being transferred to the research team.

Data on the numbers of HbA1c and cholesterol tests will be extracted from the main laboratory computer information system for each referring general practice for a period of 12 months before (to provide baseline values). Outcome data for these variables will be collected following a period of 18 months after the start of the phase one interventions.

### Assessment of intervention fidelity

We will contact different practices in each of the four study arms over regular intervals to check whether practices are receiving their randomly allocated messages, and that the messages are continuing over the intervention period as planned.

### Sample size

The sample size calculations, based on methods described by Donner *et al*. [12], were undertaken using a programme developed by Campbell *et al*. [13]. They were originally based upon the following assumptions: The study would be carried out in a population of 39 practices with an average number of 62 patients per practice (patients with diabetes whose care is undertaken either by the general practice or shared between the general practice and hospital); a significance level of 5%; 80% power; for binary outcomes (*e.g*., was a test ordered?) the intraclass correlation coefficient (ICC) equals 0.2; and for continuous outcomes (*e.g*., mean HbA1c) the ICC = 0.05.

Under these assumptions, we will be able to detect a 20% improvement (from 55% to 75%) in a binary outcome measure and an effect size of 0.24 in a continuous outcome measure. This represents a change of 0.35% in mean HbA1c, 0.26 mmol/L in mean cholesterol, 4.78 mmHg in mean systolic blood pressure and 2.45 mmHg in mean diastolic blood pressure. These were based on estimated standard deviations of 1.45, 1.07, 19.9 and 10.2, respectively, from a recent trial of a diabetes recall and management system for primary care [14].

When we subsequently extended the evaluation, we kept the same basic assumptions for the power calculation, with the exception that the number of participating practices had fallen to 34. We will be able to detect a 21% improvement (from 55% to 76%) in a binary outcome measure and an effect size of 0.25 in a continuous outcome measure. This represents a change of 0.36% in mean HbA1c, 0.27 mmol/L in mean cholesterol, 4.98 mmHg in mean systolic blood pressure and 2.55 mmHg in mean diastolic blood pressure.

The statistical power for the evaluation of the foot examination message is problematic. Our original assumptions during the planning of Phase Two had been based on pre-intervention compliance much lower than the 90% suggested by recently available QOF data. Based on the preceding assumptions (including 34 practices), we have only 58% power to detect a 5% improvement (from 90% to 95%).

### Statistical analysis

For Phase One, the dependent variables will be the last recorded HbA1c and cholesterol levels. For Phase Two, the dependent variables will be the last recorded levels of diastolic and systolic BP and the proportions of patients with recorded foot inspections.

Subjects included in the analysis will be all those patients registered with each practice with a diagnosis of diabetes whose care is undertaken either by the general practice or shared between the general practice and hospital. Where multiple observations (*e.g*., of cholesterol levels) are available for one subject within a pre- or post-intervention period, we will use the most recent observation. Data will be analysed using multilevel modelling with patients nested within practices. Within practices, a binomial error structure will be assumed for binary variables and a normal error structure for continuous variables. In each case, variation between practices will be fitted as a random effect with a Gaussian distribution.

The dependent variable will be the outcome measure corresponding to the period after the intervention. Where appropriate, the pre-intervention value of the variable will be included in the model as a covariate. The difference between practices receiving a particular intervention and those not receiving that intervention will be included as a fixed effect.

We will assess any change in blood pressure across all participants but fit a different effect for patients with microalbuminuria at baseline. This analysis will allow us to determine whether the messages had a differential effect according to whether microalbuminuria is present.

The revised study can be conceptualised as four separate trials of different education messages with each message being targeted at a specific outcome. The first step in the analysis will be to obtain estimates of the effect of each intervention on the primary outcome with which it is associated. The second step will be to estimate the effect of each intervention on the secondary outcomes with which it is associated. The final step will be to investigate whether an intervention has had a secondary effect on outcomes other than those targeted. The interventions have been introduced two at a time; allocation of practices to interventions within each pair was according to a two-by-two factorial design. Estimates of the secondary effects of an intervention will be based on a main effects model that also includes the effect of the intervention (introduced at the same time) which targets that particular outcome.

### Ethical review

Ethical approval for the study was obtained from the Newcastle and North Tyneside Research Ethics Committee.

## Competing interests

Martin Eccles is Co-Editor in Chief of Implementation Science and Robbie Foy is Associate Editor; all editorial decisions on this article were made by Co-Editor in Chief Brian Mittman.

## Authors' contributions

ME and GH conceived the original idea for this study. RF wrote the first draft of the protocol and all authors participated in discussions concerning its development and applications for grant funding. All authors contributed to revisions of the manuscript, and read and approved the final manuscript.

## Supplementary Material

Additional file 1 CONSORT checklist. Checklist indicating compliance with CONSORT criteria for reporting of randomised controlled trials. Click here for file
